# Role of CYP2E1 polymorphisms in breast cancer: a systematic review and meta-analysis

**DOI:** 10.1186/s12935-016-0371-9

**Published:** 2017-01-07

**Authors:** Yu Lu, Xuan Zhu, Cuiping Zhang, Kongmei Jiang, Chunni Huang, Xue Qin

**Affiliations:** Department of Clinical Laboratory, First Affiliated Hospital of Guangxi Medical University, Nanning, 530021 Guangxi China

**Keywords:** Breast cancer, CYP2E1, Enzyme, Polymorphism, Meta-analysis

## Abstract

**Background:**

CYP2E1 polymorphisms have been reported to influence individual’s breast cancer susceptibility as a phase I enzyme, but the results of these previous studies remain controversial. We performed a comprehensive meta-analysis to assess their association.

**Methods:**

A comprehensive search of literature included in various databases (PubMed, Web of Science and Google scholar), published before August 2016, was performed. Odds ratios (ORs) with 95% confidence intervals (CIs) calculated in fixed or random-effects models were used to estimate the strength of the associations between three polymorphisms of CYP2E1 and breast cancer susceptibility. Subgroup analysis, sensitivity analysis and test for publication bias were also performed. A total of 11 separate comparisons involving 4311 cases and 4407 controls were included in the meta-analysis.

**Results:**

Our result showed that there was no significant association between the two common polymorphisms CYP2E1 rs2031920 *C*>*T*, CYP2E1*5 *Rsa I/Rst I (c1/c2)* and BC risk. For CYP2E1*6 *Dra I (D/C)* polymorphism, a significantly increased BC risk in the overall population was found in genetic model D/C vs. D/D (OR = 1.29, 95% CI = 1.04–1.61, P = 0.023) and C/C + D/C vs. D/D (OR = 1.25, 95% CI = 1.04–1.51, P = 0.019), together with subjects who have at least one C allele (C vs. D: OR = 1.46, 95% CI = 1.20–1.79, P < 0.001). Similar results were also found in subgroup analyses in Caucasians of these three comparison models.

**Conclusions:**

The present meta-analysis suggests that CYP2E1*6 *Dra I (D/C)* variation significantly associated with the risk of BC. Individuals with D/C and C/C + D/C genotypes or carried at least one C allele of CYP2E1*6 *Dra I (D/C)* polymorphism had a significant higher susceptibility to develop BC.

**Electronic supplementary material:**

The online version of this article (doi:10.1186/s12935-016-0371-9) contains supplementary material, which is available to authorized users.

## Background

Worldwide, breast cancer (BC) represents the leading cause of female gynecological cancer death. Its estimated deaths (189,000) were reported almost equal to the estimated number of deaths from lung cancer (188,000 deaths) [1], indicating that BC has become a global burden. Known risks factors contribute to BC included reproductive events, hormonal level, and family histories [2], but they account for less than 47% cases [3]. Other etiology, though remains unknown, is believed causing by an integrated function of carcinogen exposure and polymorphisms in genes, especially genes involved in carcinogen metabolism [4].

Cytochrome P4502E1 (CYP2El), a member of Cytochrome P450 (CYP) super-family which involved in the metabolism of many endogenous and exogenous substance, is of pivotal importance in metabolizing ethanol and low-molecular-weight carcinogens such as *N*-nitrosamines in cigarettes [5, 6]. Alcohol intake, a risk factor for cancer of various organs, has been proved associated with BC [7]. The relationship between tobacco smoke and BC development, also continuously, been emphasized that smoking could increased breast cancer risk, no matter passively or actively [8]. Furthermore, their association with BC according to carcinogen-metabolizing genotype was also investigated by more than 50 epidemiologic studies. Results indicating that gene polymorphisms, including CYP450s, glutathione *S*-transferases, *N*-acetyltransferases, and sulfotransferases, interacting with carcinogen exposure, may modified one’s susceptibility to cancer [9]. Among the various CYPs, CYP2E1 is an important Phase I enzyme involved in the metabolism of alcohol and tobacco-generated *N*-nitrosamines, altering its activity has been suggested might link to the development of BC [4].

CYP2E1 is located at chromosome 10. So far, more than 100 single nucleotide polymorphisms (SNPs) have been found (http://www.ncbi.nlm.nih.gov/SNP). However, only several common mutations were extensively investigated as they might alter the activity of CYP2E1 [10, 11]. Rs3813867 *G*>*C* and rs2031920 *C*>*T* were two key SNPs among them, with the former one associated with Pst I restriction enzyme site and the later one with Rsa I restriction enzyme site, their linkage disequilibrium also lead to the CYP2E1*5 haplotype and form three types of distinct genotypes: (1) *c1/c1(Rsa I*+ *Pst I*−*)*, homozygous of normal alleles; (2) *c1/c2*, heterozygous; (3) *c2/c2 (Rsa I*− *Pst I*+*)*, homozygous alleles after nucleotide exchanged [12]. Another polymorphism, recognized by Dra I restriction enzyme in intron 6 (rs6413432), form CYP2E1*6 polymorphism and also result in three genotypes: *C/C, C/D and D/D* [13].

The relationship between above CYP2E1 polymorphisms and BC has been investigated by various studies, however, presenting conflict results. One studies conducted on patients suffering from primary unilateral BC demonstrated the absence of any association between CYP2E1*5 polymorphisms with BC, no matter in premenopausal or postmenopausal women [14]. However, Wu et al. [15], who carried out a studies on non smoker and non drinker women, reported that individuals with the *c2/c2* genotype of CYP2E1*5 had a lower BC risk than that of *c1/c1* (OR = 0.24, 95% CI = 0.08–0.74). While the most recently study by Chong et al. [16]. indicated that the c1/c2 genotype or c2 allele carriers with CYP2E1*5 variation have an approximately 1.8-fold higher risk of BC. Such controversy results may due to the relatively low mutation frequency of CYP2E1 and small epidemiologic studies with low statistic power; we therefore systematically reviewed and performed a meta-analysis to quantitatively evaluate the role of CYP2E1 polymorphisms in BC development.

## Methods

### Search strategy

A comprehensive search of literature listed in various databases (PubMed, Web of Science and Google scholar), published before August 2016, was performed using the following key words ‘breast cancer’ or’ ‘breast carcinoma’, ‘polymorphism’ or ‘variant’ and ‘mutation’, all combined with Medical Subject Heading (MeSH) term ‘CYP2E1’. The eligible studies were retrieved, and their reference lists were screen by hand to find every relevant paper. No any restriction such as time and language was made during the searches, as well as attempts to obtain unpublished studies.

### Selection criteria

In this study, we performed the meta-analysis according to the proposal of Meta-analysis of Observational Studies in Epidemiology group (MOOSE) [17]. The eligible studies were requested to meet the following inclusion criteria: (1) any type of comparative study; (2) evaluated the association between CYP2E1 gene polymorphisms and breast cancer risk; (3) in cases and controls, provided sufficient data to estimate the odds ratio (OR) with their 95% confidence intervals (95% CIs). Studies were excluded if one of them existed: (1) insufficient data to extract; (2) without control population; and (3) some CYP2E1 polymorphisms that rarely reported. If overlapping data was found, either the study with lower quality or the earlier published one would be excluded in the following analysis.

### Data extraction

Data extraction from each eligible study was conducted by two independent investigators, which included: (1) the first author’s name; (2) year of publication; (3) study region or country; (4) ethnicity; (5) cancer confirmation; (6) sample size (both cases and controls); (7) source of control (together with matching criteria); (8) polymorphisms of CYP2E1; (9) genotyping method; (10) genotype distribution in cases and controls and whether P value of the control population consistent with the Hardy–Weinberg equilibrium (HWE). In the event of different results, discussion was conducted to solve the discrepancies. When a study reported the results on different CYP2E1 polymorphisms, we treated them as separate studies in our meta-analysis.

### Quality assessment

To evaluate the quality of the included studies, a set of predefined criteria originally proposed by Thakkinstian et al. [18]. was used. The predefined criteria, which cover the credibility of controls, the representativeness of cases, specimens of cases when determining genotypes, Hardy–Weinberg equilibrium in controls, and total sample size, was structured as a 16-item list with scores ranging from 0 to 15 by Qin et al. [19]. and has been quoted by several meta-analyses [20, 21] (see Additional file 1: Table S1). As done previously, the studies with scores ≥10 were defined as high-quality studies, while the rest were low-quality studies.

### Statistical analysis

The association of each CYP2E1 polymorphisms with breast cancer risk was estimated by calculating pooled ORs and 95% CIs under different comparison models, including additive models, recessive model, and dominant model. Firstly, the heterogeneity between studies would be assessed by the Q test and I^2^ statistics. According to the presence (P_Q_ < 0.1 or I^2^ ≥ 50%) or absence (P_Q_ ≥ 0.1 and I^2^ < 50%) of heterogeneity, different models would be used to calculate the pooled ORs, with the former situation using DerSimonian–Laird random-effects model while the later using Mantel–Haenszel fixed-effects model. If heterogeneity existed, Galbraith plot analyses would be carried out to investigate the sources of heterogeneity among studies. Then, subgroup analysis by ethnicity would be performed to address possible effects of these polymorphisms on different population. To assess the stability of the results, sensitivity analysis was performed by sequential omission of individual studies, especially studies whose genotype frequencies in the control populations were deviated from HWE, as they may generate bias. HWE in the control group population, if not reported in the original article, would be tested via a goodness-of-fit Chi square test. Finally, for each polymorphism, the Begg’s funnel plots and Egger’s linear regression test was used to test the publication bias (P < 0.05 indicated a significant publication bias). All analyses were performed with Stata software (Stata/SE version 12.0, Stata Corp, College Station, TX) and all P values were two-sided.

## Results

### Study characteristics

**Fig. 1:**
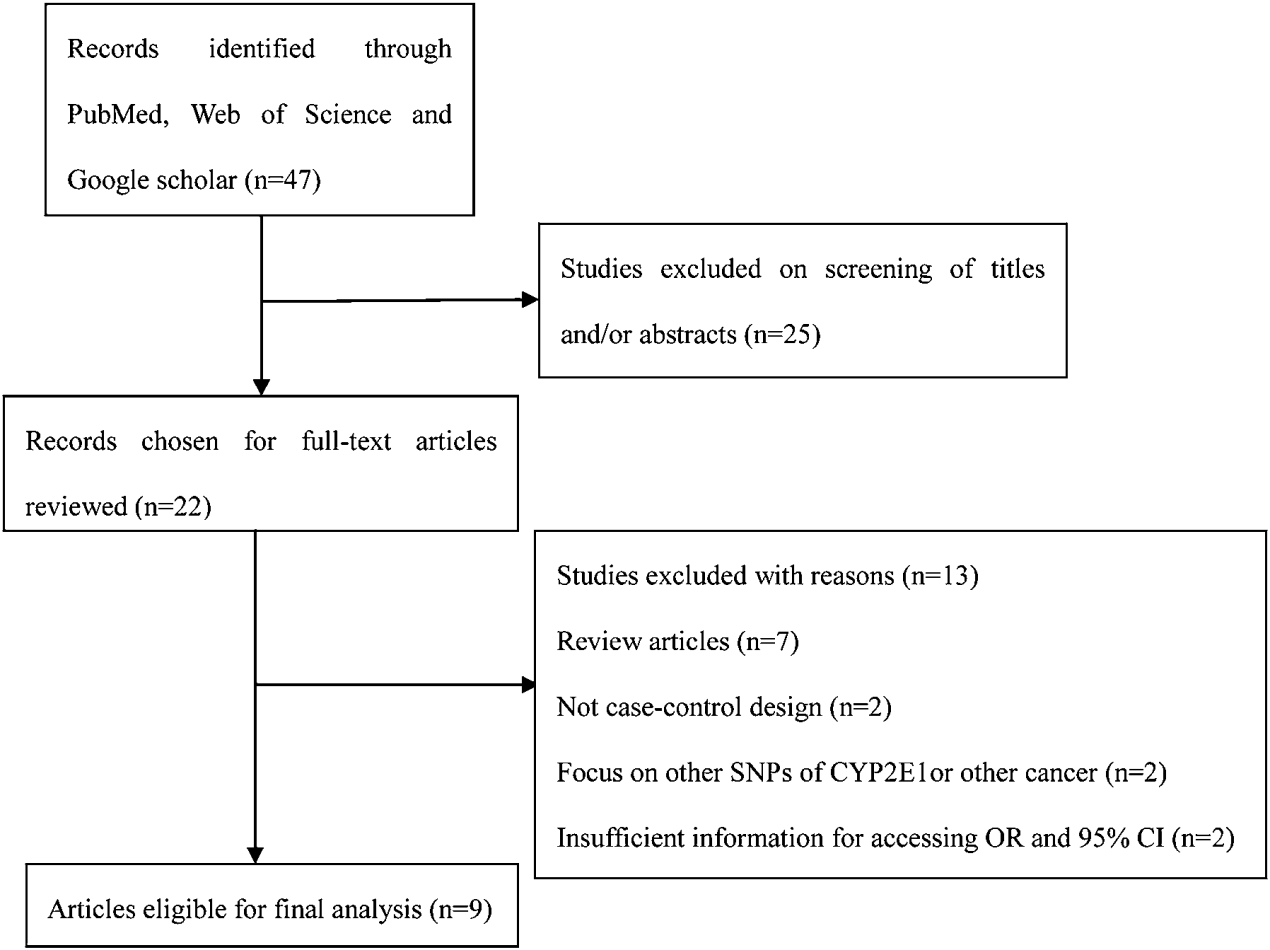
Schematic representation of study selection procedure

There were 47 published articles relevant to the search terms. By browsing the title and abstract, 25 studies were excluded because of obvious irrelevance. After a careful full-text review of the remaining 22 studies, a further 13 articles were removed: 7 were reviews; 2 were not case–control studies; 2 focus on other SNPs of CYP2E1 or other cancer; and the rest 2 did not report sufficient data. Additional eligible studies were not found through manual search of the reference lists. Consequently, night case–control studies focus on three CYP2E1 polymorphisms (rs2031920 *C*>*T*, CYP2E1*5 *Rsa I/Pst I* and CYP2E1*6 *Dra I*) and breast cancer risk were included in our meta-analysis [14, 15, 22–27]. Among them, the study by Zgheib et al. [25]. and Chong et al. [16]. explored the relationship of CYP2E1 mutation and breast cancer risk in both CYP2E1*5 and CYP2E1*6 polymorphisms, and were treated independently. As a result, a total of 11 separate comparisons involving 4311 cases and 4407 controls were finally included in the meta-analysis. A schematic representation showing the process of inclusion/exclusion of studies was illustrated in Fig. 1.

Of all the selected articles, three studies consist of 1915 cases and 1793 controls evaluated the association of CYP2E1 polymorphisms and breast cancer risk in rs2031920 *C*>*T* polymorphism (one in African, one in Asian and one in a mixed population) [14, 22, 23]; another four studies, with a total of 906 cases and 961 controls, was about CYP2E1*5 *Rsa I/Pst I* polymorphism (three in Asian and one in Arab) [15, 24, 25]; the rest four studies, including 1490 cases and 1653 controls, focus on CYP2E1*6 *Dra I* polymorphism (two in Caucasian, one in Arab and one in Asian) [25–27]. Among them, cases were major confirmed pathologically (8 studies) with their genotype determined using PCR–RFLP assays (7 studies), the rest were histologically confirmed or not mentioned with genotyping via standard PCR methods or TaqMan™ assays. The genotype distributions of the controls in two studies were found to deviate from HWE in rs2031920 *C*>*T* polymorphism while others were all reported or calculated consistence with HWE. All studies included met quality criteria ranging from 9 to 14, hence two studies were regarded as low-quality and night was high-quality. Basic characteristics of all eligible studies were listed in Table 1.

**Table 1:** Basic characteristics of all eligible studies in the meta-analysis

Author, year	Region	Ethnicity	Case/control	BC confirmation	Genotyping method	Source of control	PI	HWE (yes/no)	QS
Khedhaier, 2008	Tunisia	African	304/244	HC	PCR–RFLP	H-B	rs2031920 C>T	Yes	13
Sangrajrang, 2010	Thailand	Asian	570/479	PC	PCR	H-B	rs2031920 C>T	No	9
McCarty, 2012	Thailand	Mix	1041/1070	PC	TaqMan™	P-B	rs2031920 C>T	No	9
Choi, 2003	Korea	Asian	346/377	HC	PCR	H-B	CYP2E1*5 Rsa I/Rst I	Yes	11
Wu, 2006	Taiwan	Asian	262/225	PC	PCR–RFLP	H-B	CYP2E1*5 Rsa I/Rst I	Yes	11
Zgheib, 2013	Lebanese	Arab	227/99	PC	PCR–RFLP	H-B	CYP2E1*5 Rsa I/Rst I	Yes	10
Chong, 2016	Malaysian	Asian	71/260	PC	PCR–RFLP	H-B	CYP2E1*5 Rsa I/Rst I	Yes	11
Shields, 1996	New York, Niagara and Erie	Caucasian	272/334	PC	PCR–RFLP	P-B	CYP2E1*6 Dra I	Yes	14
Anderson, 2012	Canada	Caucasian	920/960	NM	PCR	P-B	CYP2E1*6 Dra I	Yes	12
Zgheib, 2013	Lebanese	Arab	227/99	PC	PCR–RFLP	H-B	CYP2E1*6 Dra I	Yes	10
Chong, 2016	Malaysian	Asian	71/260	PC	PCR–RFLP	H-B	CYP2E1*6 Dra I	Yes	11

*BC* breast cancer, *HC* histologically confirmed, *PC* pathologically confirmed, *NM* not mentioned, *PCR* polymerase chain reaction, PCR–RFLP polymerase chain reaction–restriction fragment length polymorphism, *HB* hospital-based, *PB* population-based, *PI* polymorphism(s) investigated, *HWE* Hardy–Weinberg equilibrium, *QS* quality score

### Meta-analysis results

**Fig. 2:**
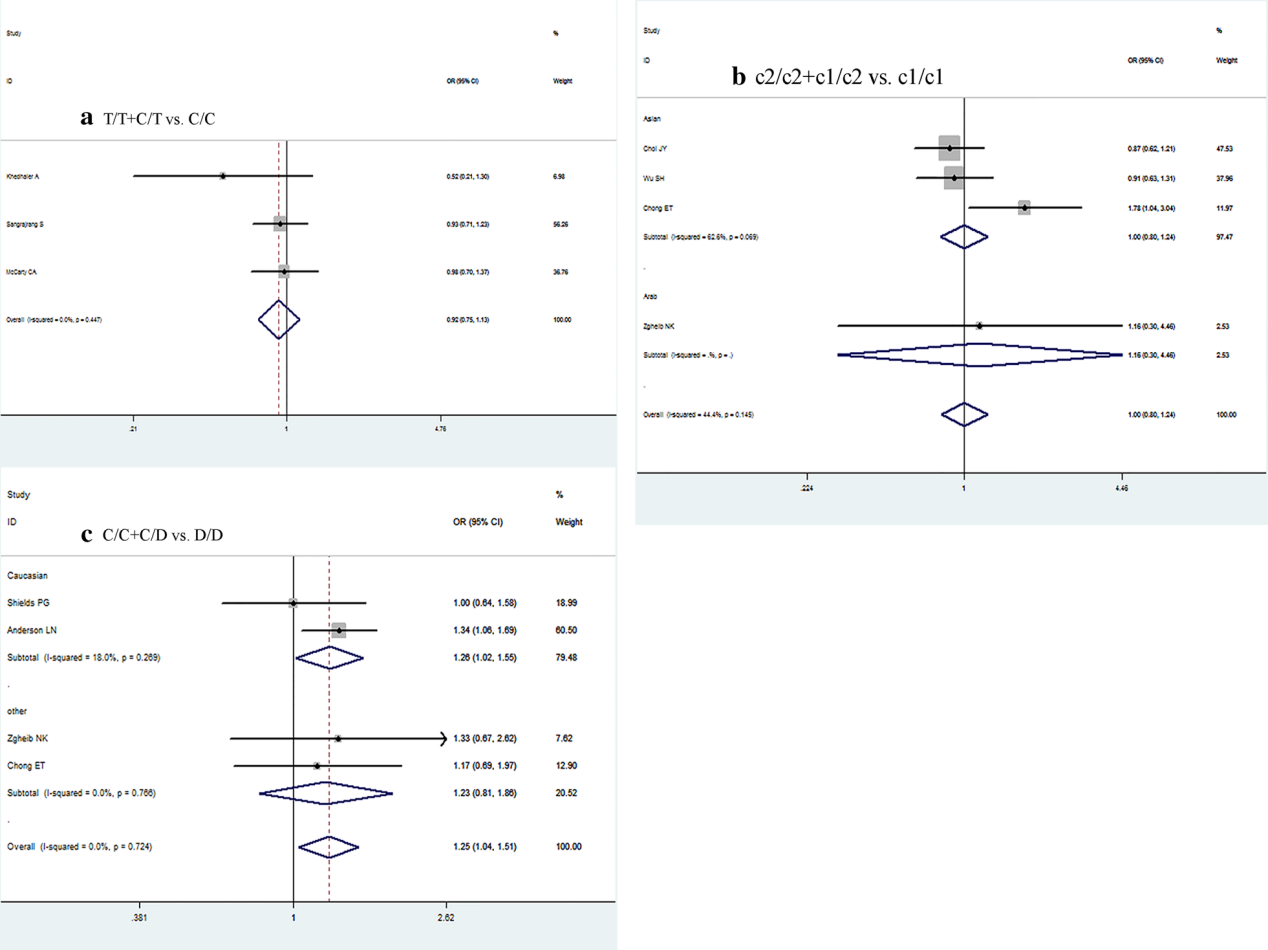
Forest plots of CYP2E1 gene polymorphisms and breast cancer (BC) risk. **a** Forest plots of CYP2E1 rs2031920 C>T polymorphism and BC risk (contrast *T/T* + *C/T* vs. *C/C*). **b** Forest plots of CYP2E1*5 *Rsa I/Rst I (c1/c2)* polymorphism and BC risk (contrast *c2/c2* + *c1/c2* vs. *c1/c1*). **c** Forest plots of CYP2E1*6 *Dra I (D/C)* polymorphism and BC risk (contrast *C/C* + *D/C* vs. *D/D*); all using a fix-effect model

The meta-analysis suggested that the rs2031920 *C*>*T* polymorphism was not associated with BC risk in all genetic models in the overall populations: (1) *T* vs. *C* (OR = 0.91, 95% CI = 0.75–1.10, P = 0.317); (2) *T/T* vs. *C/C* (OR = 0.87, 95% CI = 0.27–2.87, P = 0.821); (3) *C/T* vs. *C/C* (OR = 0.94, 95% CI = 0.76–1.16, P = 0.549); (4) *T/T* + *C/T* vs. *C/C* (OR = 0.92, 95% CI = 0.75–1.13, P = 0.434); (5) *T/T* vs. *C/T* + *C/C* (OR = 0.87, 95% CI = 0.26–2.89, P = 0.825) (Fig. 2a). Moreover, no subgroup analysis was conducted in this mutation due to all studies included were carried out in different ethnicity. Considering the significant heterogeneity found in *T/T* vs. *C/C* and *T/T* vs. *C/T* + *C/C*, random-effects model were used in these comparison model, while the rest using fix-effects model.

For the CYP2E1*5 *Rsa I/Pst I (c1/c2)* polymorphism, we still failed to identify any significant association with BC susceptibility: (1) *c2* vs. *c1* (OR = 0.97, 95% CI = 0.80–1.17, P = 0.718); (2) *c2/c2* vs. *c1/c1* (OR = 0.74, 95% CI = 0.42–1.30, P = 0.300); (3) *c1/c2* vs. *c1/c1* (OR = 1.03, 95% CI = 0.81–1.29, P = 0.797); (4) *c2/c2* + *c1/c2* vs. *c1/c1* (OR = 1.00, 95% CI = 0.80–1.24, P = 0.994); (5) *c2/c2* vs. *c1/c2* + *c1/c1* (OR = 0.75, 95% CI = 0.43–1.30, P = 0.303) (Fig. 2b). Subgroup analysis, focus on Asian population due to the limited number of included studies, also found null association in all comparison models. As no obvious heterogeneity was observed, fix-effects model was used to pool all comparison data of this polymorphism.

With regard to the CYP2E1*6 *Dra I (D/C)* variation, our result indicated a significant increased BC risk in genetic model *D/C* vs. *D/D* (OR = 1.29, 95% CI = 1.04–1.61, P = 0.023) and *C/C* + *D/C* vs. *D/D* (OR = 1.25, 95% CI = 1.04–1.51, P = 0.019) (Fig. 2c), as well as in allele model *C* vs. *D* (OR = 1.28, 95% CI = 1.05–1.55, P = 0.014). When stratified by ethnicity, similar results were also found in Caucasians in these three comparison models. Details were present in Table 2. Because no significant heterogeneity existed among all the comparison models, fix-effects model were used.

**Table 2:** Meta-analysis of the CYP2E1 gene polymorphisms on breast cancer risk

Comparison	Population	No. of studies	Test of association		Mode	Heterogeneity	
			OR (95% CI)	*P* value		I^2^ (%)	*P* value
rs2031920 *C*>*T*							
T vs. C	Overall	3	0.91 (0.75–1.10)	0.317	F	0.0	0.380
T/T vs. C/C	Overall	3	0.87 (0.27–2.87)	0.821	R	64.0	0.096
C/T vs. C/C	Overall	3	0.94 (0.76–1.16)	0.549	F	0.0	0.414
T/T + C/T vs. C/C	Overall	3	0.92 (0.75–1.13)	0.434	F	0.0	0.447
T/T vs. C/T + C/C	Overall	3	0.87 (0.26–2.89)	0.825	R	64.3	0.094
CYP2E1*5 *Rsa I/Pst I (c1/c2)*							
c2 vs. c1	Overall	4	0.97 (0.80–1.17)	0.718	F	45.9	0.136
	Asian	3	0.96 (0.80–1.16)	0.693	F	63.5	0.065
c2/c2 vs. c1/c1	Overall	4	0.74 (0.42–1.30)	0.300	F	49.7	0.137
	Asian	3	0.74 (0.82–1.30)	0.300	F	49.7	0.137
c1/c2 vs. c1/c1	Overall	4	1.03 (0.81–1.29)	0.797	F	40.8	0.167
	Asian	3	1.31 (0.74–2.34)	0.823	F	60.3	0.080
c2/c2 + c1/c2 vs. c1/c1	Overall	4	1.00 (0.80–1.24)	0.994	F	44.4	0.145
	Asian	3	1.00 (0.80–1.24)	0.965	F	62.6	0.069
c2/c2 vs. c1/c2 + c1/c1	Overall	4	0.75 (0.43–1.30)	0.303	F	48.0	0.146
	Asian	3	0.75 (0.43–1.30)	0.303	F	48.0	0.146
CYP2E1*6 *Dra I (D/C)*							
C vs. D	Overall	4	1.28 (1.05–1.55)	*0.014*	F	0.0	0.532
	Caucasian	2	1.32 (1.06–1.64)	*0.014*	F	–	–
C/C vs. D/D	Overall	4	1.47 (0.75–2.91)	0.266	F	0.0	0.667
	Caucasian	2	1.66 (0.68–4.09)	0.268	F	–	–
D/C vs. D/D	Overall	4	1.29 (1.04–1.61)	*0.023*	F	0.0	0.667
	Caucasian	2	1.32 (1.04–1.68)	*0.025*	F	–	–
C/C + D/C vs. D/D	Overall	4	1.25 (1.04–1.51)	*0.019*	F	0.0	0.724
	Caucasian	2	1.26 (1.02–1.55)	*0.032*	F	18.0	0.269
C/C vs. D/D + D/C	Overall	4	1.39 (0.71–2.72)	0.339	F	0.0	0.654
	Caucasian	2	1.58 (0.64–3.89)	0.318	F	–	–

*F* fixed-effects model, *R* random-effects model

Italic values indicate significant difference (p < 0.05)

### Heterogeneity analysis

**Fig. 3:**
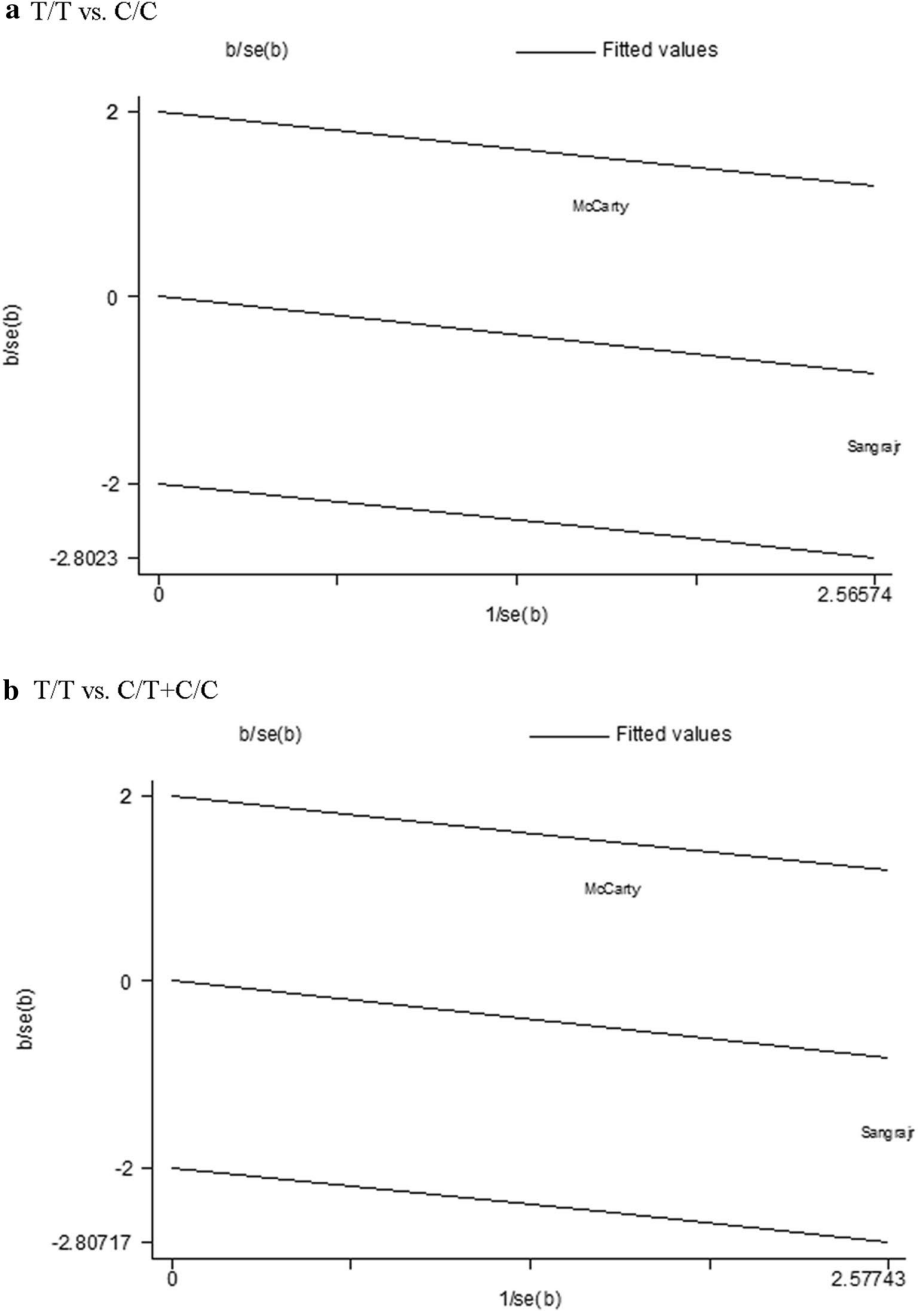
Galbraith plots of CYP2E1 gene polymorphisms and breast cancer (BC) risk in comparison models with significantly heterogeneity. **a** *T/T* vs. *C/C* in rs2031920 C>T polymorphism. **b** *T/T* vs. *C/T* + *C/C* in rs2031920 C>T polymorphism

As significant heterogeneity was found in the *T/T* vs. *C/C* and *T/T* vs. *C/T* + *C/C* comparison models of rs2031920 *C*>*T* polymorphism, Galbraith plot analyses were carried out to detect the possible source of heterogeneity. However, as show in Fig. 3, outliner was observed in neither *T/T* vs. *C/C* model nor *T/T* vs. *C/T* + *C/C* model, indicating that the studies included in both two comparison models were not contributors to the heterogeneity.

### Sensitivity analysis

Considering that there were two studies whose genotypes inconsistent with HWE in rs2031920 *C*>*T* variation, sensitivity analysis were performed to see if any single study would greatly influenced the estimates of overall risk, our result showed that the pooled ORs did not materially altered with or without these studies (data not shown). But we could not conduct sensitivity analysis for *T/T* vs. *C/C* model and *T/T* vs. *C/T* + *C/C* model due to a limited study number in these two models (only three studies were included and one of them did not provided data in T/T genotype).

### Publication bias

**Fig. 4:**
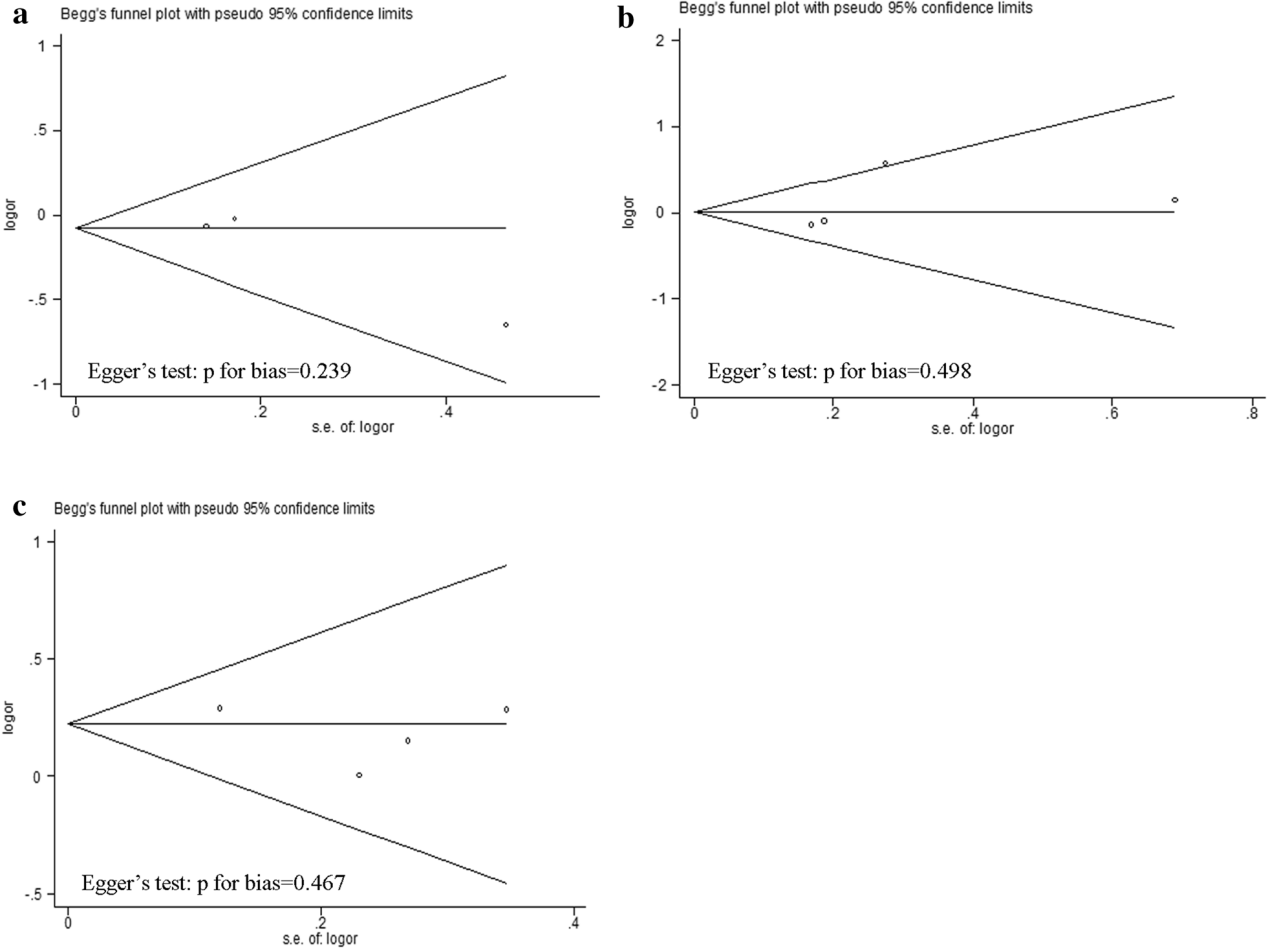
Begg’s funnel plot analysis and Egger’s test to detect publication bias. Each *point* represents a separate study for the indicated association. **a** Begg’s funnel plot analysis and Egger’s test for contrast *T/T* + *C/T* vs. *C/C*. **b** Begg’s funnel plot analysis and Egger’s test for contrast *c2/c2* + *c1/c2* vs. *c1/c1*. **c** Begg’s funnel plot analysis and Egger’s test for contrast *C/C* + *D/C* vs. *D/D*

To assess possible publication bias, Begg’s funnel plots and Egger’s tests were performed simultaneously. The funnel plots were symmetrical in all genetic models of three CYP2E1 polymorphisms, indicating no significant publication bias existed in all the articles included. Egger’s test, with all the P value larger than 0.05, also revealed no evidence of publication bias in our meta-analysis (Fig. 4).

## Discussion

Breast cancer, of which heredity explains approximately 10–15% of the cases, with only 5% can be clarified by known genetic polymorphisms such as BRCA1 and BRCA2 [28]. Such fact suggesting that other potential, common, but low-penetrance genetic variants may contribute to individual’s susceptibility to breast cancer. CYP2E1, a Phase I enzyme responsible for the metabolic activation of various carcinogens such as *N*-nitrosaminesan and alcohol, was in different activity among individuals [12]. It has been assumed that polymorphisms of CYP2E1*5 and CYP2E1*6 may lead to a decreased activity in CYP2E1 enzyme, thus linked to a lower risk of cancer. Nevertheless, the power of a single study was too small to draw a precise conclusion, we therefore investigated breast cancer and CYP2E1 polymorphisms in these common mutations using a meta-analysis.

However, in the present study, no significant association was found between SNP rs2031920 *C*>*T* polymorphism and BC. The haplotype CYP2E1*5, which consist of SNP rs2031920 *C*>*T* and 3813867 *G*>*C*, also failed to identify any significant association with BC risk. Single SNP rs3813867 *G*>*C* was also taken into consideration, but further analysis was not carried out due to the suboptimal study numbers (n = 1) [27]. Such insignificant results may be partially attributed to the different distribution of the CYP2E1*5 polymorphism between varies races, with the rarest 0.05 in Caucasians and the highest 0.23 in oriental populations [12]. Nevertheless, after stratified the study populations into different races, where we mainly focus on Asian populations, the results still failed to indicate any association between CYP2E1*5 polymorphism and BC development. The pooled results of rs2031920 *C*>*T* SNP and BC risk were consistent with those studies included in the present meta-analysis, all indicating an insignificant relationship between them; and the overall results of CYP2E1*5 polymorphism and BC were also accordance with half of those included, though one of the rest observed a decreased risk of BC while another revealed an increased risk. Taken together, it may be concluded that CYP2E1*5 polymorphisms are not associated with BC risk in the overall population.

Interestingly, when considering the CYP2E1*6 polymorphism, our study found that individuals with the *D/C* and *C/C* + *D/C* genotype had a significantly higher risk of BC compared to those with the *D/D* genotype, similar increased result was also found in the C allele carriers when compared with the D allele carriers, especially in Caucasian population. These results suggested that polymorphism in CYP2E1*6 could be a risk factor for BC development. But such result was inconsistent with those of the original studies, of which all suggested no significant relationship between any comparison model of CYP2E1*6 variation and BC development.

Actually, our results should explain with caution as there is increasing evidence that metabolizing enzymes do not act alone. In the study carried out by Choi et al. [24]. that explored the role of alcohol and genetic polymorphisms of CYP2E1*5 in BC development, no significant overall differences were found in the *c1/c2* genotype frequencies between BC cases and controls. However, after taking the drinking situation into consideration, a 1.9-fold increasing risk for developing BC was found when comparing the ‘ever’-drinking women with the c2 mutation to the non-drinkers with the *c1/c1* mutation. Another study, investigated lifetime passive cigarette smoke exposures together with genetic variants and BC risk in women who had never smoked, found that interaction between passive smoke exposure and CYP2E1*6 *AA/AT* (namely *CC/CD*) polymorphism could significant increased breast cancer risk among premenopausal women [27]. In sum, such gene-environment interaction should be taken into consideration when investigating CYP2E1 polymorphism in the development of BC, however, due to the limited studies included, our study could not conduct further analysis with these factors taken into consideration.

To our knowledge, this is the first meta-analysis carried out to date to evaluate the role of CYP2E1 polymorphisms in breast cancer susceptibility. Despite the findings mentioned above, this study had several limitations. First, we haven’t taken the gene-environment interaction into consideration. As is known to all, apart from genetic factors, smoking status and alcohol consumption are important risk factors for BC; however, we could not conduct subgroup analyses stratified by environmental exposure due to the limited information on our included studies. Second, the overall results of our study were based on crude ORs, but a more precise evaluation should be adjusted for the know risk factors such as age and menopause status. Third, the number of studies included in this study is relatively small, with three or four studies for each polymorphism, which may lead to low statistical power and prevent us from exploring a real association of the CYP2E1 polymorphism and BC risk. Fourth, because no attempts were made to access unpublished studies and studies in languages other than English, publication bias may exist, though results of our Begg’s funnel plot and Egger’s test did not reveal any publication bias. Fifth, as most studies were conducted in Asian and Caucasian population, the relative lack of ethnic diversity demands for further studies.

## Conclusions

Aside from the above limitations, this meta-analysis suggests that CYP2E1*6 *Dra I (D/C)* polymorphism might be associated with increased BC risk, individuals with D/C and C/C + D/C genotypes or carried at least one C allele of CYP2E1*6 *Dra I (D/C)* polymorphism had a significant higher susceptibility to develop BC, in Caucasians, particularly. Whereas, no any significant relationship between CYP2E1*5 *Rsa I/Rst I (c1/c2)*, rs2031920 *C*>*T* polymorphisms and BC risk was found.

## Additional file

**Additional file 1: Table S1.** Scale for quality assessment.

## Abbreviations

OR: odds ratios
CI: confidence intervals
BC: breast cancer
SNP: single nucleotide polymorphisms
HWE: Hardy–Weinberg equilibrium
