# Supplementary material for: Role of CYP2E1 polymorphisms in breast cancer: a systematic review and meta-analysis
Source: Cancer Cell Int. 2017 Jan 7;17:11. doi: 10.1186/s12935-016-0371-9 (PMC5219772; doi:10.1186/s12935-016-0371-9)
Supplement: Supplementary file 1 — Additional file 1: Table S1. Scale for quality assessment. [file 12935_2016_371_MOESM1_ESM.doc]

Table S1 Scale for quality assessment

| Criteria | Score |
| --- | --- |
| Representativeness o f cases |  |
| Selected from population or disease registry | 3 |
| Selected from hospital | 2 |
| Selected from pathology archives, but without description | 1 |
| Not described | 0 |
| Credibility of controls |  |
| Population- based | 3 |
| Blood donors or volunteers | 2 |
| Hospital-based | 1 |
| Not described | 0 |
| Specimens of cases determining genotypes |  |
| White blood cells or normal tissues | 3 |
| histopathology or exfoliated cells of tissue | 0 |
| Hardy-Weinberg equilibrium in controls |  |
| Hardy-Weinberg equilibrium | 3 |
| Hardy-Weinberg disequilibrium | 0 |
| Total sample size |  |
| 》1000 | 3 |
| 》400 but <1000 | 2 |
| 》200 but <400 | 1 |
| <200 | 0 |

1. Qin X, Peng Q, Chen Z, Deng Y, Huang S, Xu J et al. The association between MTHFR gene polymorphisms and hepatocellular carcinoma risk: a meta-analysis. PLoS One. 2013;8(2):e56070. doi:10.1371/journal.pone.0056070.
